# Identifying paediatric nursing-sensitive outcomes in linked administrative health data

**DOI:** 10.1186/1472-6963-12-209

**Published:** 2012-07-20

**Authors:** Sally Wilson, Alexandra P Bremner, Yvonne Hauck, Judith Finn

**Affiliations:** 1 School of Population Health, The University of Western Australia, 35 Stirling Hwy, Perth, Western, Australia; 2 School of Nursing and Midwifery, Curtin Health Innovation Research Institute (CHIRI), Curtin University, GPO Box U1987, Perth, Western, Australia; 3 Discipline of Emergency Medicine, School of Primary, Aboriginal and Rural Health Care, The University of Western Australia, 35 Stirling Hwy, Perth, Western, Australia

## Abstract

**Background:**

There is increasing interest in the contribution of the quality of nursing care to patient outcomes. Due to different casemix and risk profiles, algorithms for administrative health data that identify nursing-sensitive outcomes in adult hospitalised patients may not be applicable to paediatric patients. The study purpose was to test adult algorithms in a paediatric hospital population and make amendments to increase the accuracy of identification of hospital acquired events. The study also aimed to determine whether the use of linked hospital records improved the likelihood of correctly identifying patient outcomes as nursing sensitive rather than being related to their pre-morbid conditions.

**Methods:**

Using algorithms developed by Needleman et al. (2001), proportions and rates of records that identified nursing-sensitive outcomes for pressure ulcers, pneumonia and surgical wound infections were determined from administrative hospitalisation data for all paediatric patients discharged from a tertiary paediatric hospital in Western Australia between July 1999 and June 2009. The effects of changes to inclusion and exclusion criteria for each algorithm on the calculated proportion or rate in the paediatric population were explored. Linked records were used to identify comorbid conditions that increased nursing-sensitive outcome risk. Rates were calculated using algorithms revised for paediatric patients.

**Results:**

Linked records of 129,719 hospital separations for 79,016 children were analysed. Identification of comorbid conditions was enhanced through access to prior and/or subsequent hospitalisation records (43% of children with pressure ulcers had a form of paralysis recorded only on a previous admission). Readmissions with a surgical wound infection were identified for 103 (4.8/1,000) surgical separations using linked data. After amendment of each algorithm for paediatric patients, rates of pressure ulcers and pneumonia reduced by 53% and 15% (from 1.3 to 0.6 and from 9.1 to 7.7 per 10,000 patient days) respectively, and an 84% increase in the proportion of surgical wound infection (from 5.7 to 10.4 per 1,000 separations).

**Conclusions:**

Algorithms for nursing-sensitive outcomes used in adult populations have to be amended before application to paediatric populations. Using unlinked individual hospitalisation records to estimate rates of nursing-sensitive outcomes is likely to result in inaccurate rates.

## Background

Nursing-sensitive outcomes are “changes in health status upon which nursing care has had a direct influence” [1,2] [p 1]. The concept arose from the quality improvement agenda of the Joint Commission on Accreditation of Health Care Organizations (JCAHO) in the United States (US) in the late 1980s [3]. More recently, concerns about the changing nurse skill mix have led researchers to investigate associations between the incidence of nursing-sensitive outcomes and levels of nurse staffing [4-8].

Based on the literature and expert clinical opinion, Needleman et al. identified 14 potential nursing-sensitive outcomes that could be measured using routinely collected administrative health data. These included: pressure ulcers, deep vein thrombosis and pulmonary embolism, pneumonia, urinary tract infection, central nervous system complications, shock or cardiac arrest, upper gastrointestinal bleed, pulmonary failure, physiologic/metabolic derangement, surgical wound infection, mortality, failure to rescue and length of stay [9]. Administrative health data are electronic records collected for administrative purposes that include patients’ hospital discharge summaries. These were determined to be the best source for constructing nursing-sensitive outcomes, because they contain diagnoses and procedures coded according to the International Classification of Diseases (ICD) and contain patient level variables such as age, sex, country of birth, and health insurance status in a relatively uniform format [9]. Needleman et al.’s development of nursing-sensitive outcomes was guided by three criteria: (1) that nursing-sensitive outcomes be conceptually related to nursing care, (2) that outcomes be ‘codable’ from hospital patient discharge (separation) abstracts, and (3) that the outcomes occur in inpatient acute care settings with high enough frequency and variation to allow for statistical analysis [9] [p 37].

For each outcome, an algorithm (syntax) was developed that used a combination of ICD-9 codes, Diagnosis Related Groups (DRGs), Major Diagnostic Categories (MDCs), length of stay, presence of a surgical procedure and age. Each algorithm included outcome specific inclusion and exclusion (qualifying) criteria in an attempt to include only patients who experienced a truly preventable adverse outcome rather than one associated with the disease process. For example, the ‘pressure ulcer’ algorithm excluded patients with any form of paralysis in their hospital discharge records to ensure those flagged with a pressure ulcer were more likely to have acquired it as a result of the quality of nursing care and it was not present on admission as a consequence of a pre-morbid condition. Algorithms excluded outcomes that were primary diagnoses, and used secondary diagnoses to identify outcomes that were potentially nursing sensitive. As the algorithms developed by Needleman et al. used American ICD-9-CM (Clinical Modification) codes, subsequent translation to ICD-10-AM (Australian Modification) codes using “crosswalks” (mapping keys) was undertaken by McCloskey [10] in New Zealand. These translated algorithms have been used in Australian studies [11,12].

Many of the challenges faced by Needleman and his team [9] in the matching of data is obviated by the data linkage processes in Western Australia (WA), whereby all patients have a unique identifier that links their individual records into a single ‘chain’ of hospitalisation episodes. Identification of comorbid conditions is enhanced because linked data allows researchers to match records of the same patient both within and across databases, thus providing longitudinal health data on individuals and populations [13-17]. Many patient hospitalisation databases only code patient conditions relevant to a specific episode of care, therefore some chronic conditions may not be recorded in a discharge abstract [9,18]. An advantage of using linked data is being able to ‘look back’ to ascertain comorbid conditions for individuals when this is not routinely recorded. Similarly, linked data enables researchers to ‘look forward’ to determine patient outcomes that require hospitalisation and are identified following discharge from the index hospitalisation. The results of a WA study reported that identification of comorbid conditions in an adult population increased from 47% using index hospitalisation data to 90% when a three year look back into administrative health data was undertaken [17]. Therefore, linked data is likely to provide more accurate information about comorbid conditions which may affect identification of nursing-sensitive outcomes.

The WA Data Linkage Branch adheres to data linkage best practice protocols [19] and uses probabilistic matching based on medical record number, surname, first name and initial, date of birth, sex and address as the principal variables to link the data. Clerical review of additional information is undertaken for records that fall between definite matches and non-definite matches [20]. Invalid and missed links have been estimated at 0.11% [21]. Validation studies have shown recording of additional diagnoses and complications in Health Morbidity Data (HMD) vary between 10-80% sensitivity depending on the nature of the condition [21].

Studies of nursing-sensitive outcomes in administrative health data have predominantly used adult populations. Some researchers have included children within their adult study populations [6,22,23]; however, few have used administrative health data of paediatric populations [24,25]. Given the differences in casemix and risk profiles between paediatric and adult patients [26,27], it is questionable whether nursing-sensitive outcomes used with adults are applicable to paediatrics. Optimal paediatric nursing care takes into account the child’s stage of development, consequently age stratification needs to be considered during analysis. Children have fewer chronic conditions and comorbidities and primary healthcare and in-home care delivery are emphasised. As a result, paediatric hospital lengths of stay are shorter than those of adults, which contributes to the challenges of studying paediatric populations. Earlier studies found that there were insufficient numbers of recorded events to make analysis meaningful for some potential nursing-sensitive outcomes [24,25]. Many comorbid conditions reported in adults are not present in children so the risks for developing adult nursing-sensitive outcomes are reduced. For the above reasons, it may not be useful to apply algorithms for nursing-sensitive outcomes that were validated in adult populations to paediatric populations. The algorithm for each outcome should be validated within paediatric populations before being used to measure the quality of paediatric nursing care.

In our earlier work with a panel of expert paediatric nurses, we established face and content validity for 17 nursing-sensitive outcomes that were potentially useful for measuring the quality of paediatric nursing using administrative health data [28]. Seven of these outcomes were also used by Needleman et al., with pressure ulcer, pneumonia and surgical wound infection ranked as top three. The present study focused on these three nursing-sensitive outcomes and aimed to determine whether Needleman et al.’s algorithms, or variations of them, were useful in paediatric populations. A further aim was to determine whether linked data provided more accurate information about comorbid conditions than using data that was not linked, and whether it affected the identification of nursing-sensitive outcomes.

## Method

This population based, retrospective cohort study used linked administrative health data from the WA HMD System which enabled ascertainment of all WA hospitalisations for the cohort. The cohort included all WA resident children admitted to one tertiary paediatric hospital during the 10 year period from July 1999 to June 2009 inclusive. Inclusion criteria were that the child had a WA postcode as place of residence, was aged ≤18 years, and had been an inpatient (stayed at least one night; determined by admission date and separation date minus days of care provided by Hospital in the Home). Children who had been transferred from another hospital were excluded and data from subsequent hospitalisation following transfers were not included. Hospital separations are only recorded when the child is discharged or transferred to another hospital. Separation records are not created when the child is transferred within the same hospital. To enable looking back for comorbid conditions and looking forward for possible consequences of the index hospitalisation, HMD from the previous ten years and all subsequent WA hospitalisations were provided for the children in the cohort. The index hospitalisation was the record in which the nursing-sensitive outcome was identified.

The linked HMD provided abstracts of demographic and clinical information on hospital separations from all acute care hospitals within WA. Up to 22 diagnostic variables and 12 procedural variables were provided. Diagnoses were coded using ICD-9-CM until June 1999, and ICD-10-AM since then. Similarly, procedures were coded according to ICD-9-CM until June 1999, but subsequently followed the Australian Classification of Health Interventions (ACHI). Variables for MDC and Australian refined (AR)-DRG were also provided.

Data were received in an anonymised file and analysed in SPSS for Windows (Version 19.0.0.1; 2010 IBM SPSS Chicago, Il, USA), with logical checks undertaken during data clean-up. The translation of ICD, DRG and MDC codes was checked and amendments were made as required. The syntax for each nursing-sensitive outcome algorithm had been written for use in SPSS (Finn, J. unpublished) and this was also checked and amended if necessary. The MDC codes that were used were MDC4 which are diseases and disorders of the respiratory system and MDC9 which are diseases and disorders of the skin, subcutaneous tissue and breast [18].

Month and year of birth were provided for each child. Age on admission was calculated, then categorised into developmentally appropriate age groups of neonate (1–28 days), infant (29–365 days), toddler (>1-3 years), pre-schooler (>3-6 years), school-age (>6-13 years) and adolescent (>13- ≤ 18 years). The risk pool for surgical wound infections was surgical patients only. This subset of the cohort contained patients classified as ‘surgical’ based on AR-DRG codes which were supplied by WA Data Linkage Branch. The remainder of the cohort was classified as ‘non-surgical’ and contained children coded as medical and ‘other’. [18]

### Process

Each hospital separation record was assumed to be an independent event, therefore calculations were based on hospital separations or records, rather than children, as done in other studies [7,11,24,29]. Nursing-sensitive outcomes of pressure ulcer, pneumonia and surgical wound infection were identified as per Needleman et al.’s algorithms (Table 1).

**Table 1:** Definitions of nursing-sensitive outcomes

**Nursing-sensitive outcome**	**Numerator**^**1**^	**Denominator**	**Key exclusions**
As per Needleman et al. [9]			
Pressure ulcer	Pressure ulcer (ICD^2^ L89)	All medical and surgical inpatients	MDC9-skin conditions
		LOS > =4 days	All diagnosis of hemiplegia, paraplegia,
			paralysis; cerebral palsy; (ICD > =G80 and < =G84)
Pneumonia	Aspiration, post-operative, hypostatic, bacterial, broncho and unspecified pneumonias: (ICD > =J14. and < =J15.6, J15.8 J15.9 J18.0 J18.2 J18.8 J18.9 J69.0 J95.8, J95.9)	All medical and surgical inpatients	MDC4-respiratory conditions
			Immunocompromised, AIDS; (ICD > =B20. and < =B24., > = D80. and < =D89.9, M35.9)
			All diagnoses of probable community acquired pneumonia (ICD > =J10. and < =J10.8, > = J11. and < =J11.8, =J12. and < =J12.9, J13., J15.7, J16.8, > = J17. and < =J17.8)
Postoperative pneumonia [24]	Aspiration, post-operative, hypostatic, bacterial, viral, broncho pneumonias	All surgical inpatients	
	(ICD J12. and < =J12.9, J13., > = J14. and < =J15.6, J15.7, J15.8, J15.9, > = J17. and < =J17.8, J18.0, J18.1, J18.2, J18.8, J18.9, J69.0, J95.851, J95.9)		
Surgical wound infection	Surgical wounds, including surgery post traumatic injury (ICD T79.3, T81.4 T81.41 T81.42)	All surgical inpatients	
		Total discharges only	
Recommended paediatric algorithm			
Pressure ulcer	Pressure ulcer (ICD L89)	All medical and surgical inpatients	All diagnosis of hemiplegia, paraplegia, paralysis; cerebral palsy; spina bifida. (ICD > =G80 and < =G84; > = Q05. and < =Q05.9 or > =Q07. and < =Q07.03)
		LOS > =3 days	All diagnoses of paralysis found in look back period
Pneumonia	As per Needleman et al. plus J15.7, J18.1, J69.8	All medical and surgical inpatients	MDC4-respiratory conditions
			All diagnoses of probable community acquired pneumonia (ICD as per Needleman et al.)
			Aspiration pneumonia and epilepsy (ICD J69.0, 69.8, > = G40. and < =G40.9, > = G41. and < =G41.9)
			All diagnosis of epilepsy found in look back period
Surgical wound infection	Surgical wounds, including surgery post traumatic injury plus those found in 30 day ’look forward period’ (ICD T79.3, T81.4 T81.41 T81.42)	All surgical inpatients	
		Total discharges only	

^1^All numerators are based on secondary diagnosis only except surgical wound infection in look forward period.

^2^ICD codes are all ICD-10-AM.

For each nursing sensitive outcome, frequencies of diagnostic and procedural codes listed in the index records of children with the outcome were calculated to identify any condition or procedure that might have contributed to the outcome, but had not been excluded in Needleman et al.’s algorithm. Selected index records were also examined individually along with previous records (look back) and subsequent separation records of the child to ascertain comorbid conditions which could increase risk. These comorbid conditions were then included as potential qualifying criteria in revised algorithms, which were applied to the paediatric population, and the index records of children determined to have the nursing-sensitive outcomes were identified. For example, to determine the effect of skin condition (MDC9) on the identification of children with nursing-sensitive pressure ulcers, the algorithm was run with and without the exclusion criterion of presence of a skin condition (MDC9).

Each child’s records were aggregated to ascertain the presence of a comorbid condition and, if present, check whether it was noted on the index record. Where necessary, the comorbid condition was added into the index record and algorithms for each nursing-sensitive outcome were re-run using the corrected index records. Based on the results, algorithms were written that should optimise identification of nursing-sensitive outcomes in paediatric populations (Table 1).

If a child had more than one type of nursing-sensitive outcome on the same or separate admissions they were counted as separate index hospitalisations. If a child was readmitted with the same nursing-sensitive outcome, the individual records were viewed. Clinical judgement was used to decide whether the outcome was the same episode or an independent event based on time between discharge and admission, diagnoses and procedures. If judged to be independent events, records were counted as separate index hospitalisations and if judged to be the same episode, then only the first admission was included as the index hospitalisation.

### Statistical analysis

Proportions of nursing-sensitive outcomes per 1,000 hospital separations were calculated for the three outcomes. Rates per 10,000 patient days were calculated using Needleman’s algorithms and the revised paediatric algorithms for pressure ulcer and pneumonia, but not surgical wound infection. The definition of surgical wound infection used for both numerator and denominator is ‘within 30 days of surgery’ [30] [p313]. It cannot be determined when the infection occurred in relation to the surgery from WA administrative health data nor can patients’ lengths of stay post-surgery be ascertained. Therefore, a rate based on total patient days would not provide an accurate measure of the risk pool.

Proportions and/or rates for each nursing-sensitive outcome were determined by running the algorithm with the qualifying criterion included and then rerunning it with the qualifying criterion excluded. For example, the number of children with pressure ulcers who also had a form of paralysis and the number of children with pressure ulcer and no form of paralysis were determined. For each nursing-sensitive outcome a number of qualifying criteria were tested. Estimated rate differences and 95% confidence intervals (CI) were calculated. If there was no statistically significant difference in the rates (CI included zero) or the number of records was <5, the qualifying criterion was dropped from the algorithm. Finally, percentages of records with comorbid conditions found for each nursing-sensitive outcome using look back data and nursing-sensitive outcomes using look forward data were calculated.

### Ethical considerations

The project had approval from the Human Research Ethics Committees of the study hospital and the WA Department of Health. To avoid possible identification of children, wherever numbers were less than 5 the exact number is replaced by <5.

## Results

From 1 July 1999 to 30 June 2009 a total of 129,719 hospital separations pertaining to 79,016 children met our inclusion/exclusion criteria. This increased to 517,605 hospital separations when linked with records in the look back (back to 1 July 1989) and the look forward (to 30 March 2010) periods.

Of the 129,179 hospital separations, boys comprised 57%, 32.4% were aged 6–13 years, 83% lived in the metropolitan area, 79% were ‘emergency’ admissions (as distinct from ‘elective’) and 83.4% were classified as ‘non-surgical’. The most common admission was respiratory (16.5%), followed by gastrointestinal (12.8%) and musculoskeletal (12%) (Table 2). Excluding same-day admissions, the length of hospital stay (LOS) ranged from 1–975 days, with median 2 days (inter-quartile range 1–4 days).

**Table 2:** Characteristics of the 10 year inpatient cohort

	**Total hospital separations**	
	**(n = 129719)**	
	***n***	**%**
Age group		
1-28 days (neonate)	5650	4.4
29-365 days (infant)	22114	17.0
>1-3 years (toddler)	23824	18.4
>3-6 years (preschooler)	14786	11.4
>6-13 years (school-age)	41988	32.4
>13- ≤ 18 years (adolescent)	21357	16.5
Sex		
Male	73931	57.0
Female	55788	43.0
Residence		
Metropolitan	107715	83.0
Rural	21908	16.9
Admission type		
Emergency	102699	79.2
Elective	27020	20.8
Case mix		
Non-surgical	108127	83.4
Surgical	21592	16.6
Major diagnostic category		
Respiratory	21341	16.5
Gastrointestinal	16593	12.8
Musculoskeletal	15562	12.0
Ear, nose throat and mouth	13326	10.3
Neurological	10271	7.9

### Pressure ulcers

Adhering to the qualifying criteria used by Needleman et al. in Table 1[9], over the 10 year study period, 49 hospital separation records included a code for pressure ulcer in one or more of the secondary diagnosis fields. This is equivalent to 1.39 pressure ulcers per 1,000 hospital separations and a rate of 1.3/10,000 patient days.

Table 3 shows the proportions and rates of each qualifying criterion for pressure ulcer. There were 2,751 records of children with a form of paralysis of whom seven had pressure ulcers (2.54/1,000 separations; a rate of 4.75/10,000 patient days). Additionally, 126,968 hospital separations had no record of a form of paralysis, of which 60 had pressure ulcer as a secondary diagnosis (0.47/1,000 separations; a rate of 1.13/10,000 patient days). There was a significant difference between the rates of pressure ulcers when a child had a form of paralysis recorded and when no paralysis was recorded (95% CI 1.83, 39.61). Numbers were too low for meaningful comparisons when the qualifying criteria of MDC9 (conditions of skin) were included or excluded (n < 5 pressure ulcers in children with a skin condition). Table 3 also provides details of numbers of hospital separations with pressure ulcers when length of stay was <2 or ≥2 days, <3 or ≥3 days and <4 or ≥4 days. The categories are not exclusive and ≥4 days included those that are in ≥2 days length of stay.

**Table 3:** Rates of pressure ulcers and analysis of qualifying criteria for pressure ulcer

	**No. of events**	**Total separations**	**No./1000 separations**	**Total patient days**	**Rate/10000 patient days**	**Diff between rates/10000 patient days**
						**95% CI**
As per Needleman	49	35125	1.39	376574	1.30	
**Qualifying criteria**						
Paralysis						
With	7	2751	2.54	14727	4.75	(1.83, 39.61)*
Without	60	126968	0.47	529376	1.13	
MDC9^1^ (disorders of skin or subcutaneous tissue)						
With	<5	5225	0.38	17170	1.16	−^2^
Without	>62	124494	0.52	526933	1.23	
LOS^3^						
> = 2 days	61	77471	0.79	491851	1.24	(0.45, 0.89)*
<2 days	6	52248	0.11	52252	1.15	
> = 3 days	57	52735	1.08	442379	1.29	(0.66, 1.24)*
<3 days	10	76984	0.13	101724	0.98	
> = 4 days	53	37521	1.41	396737	1.34	(0.87, 1.65)*
<4 days	14	92198	0.15	147366	0.95	
As per recommended paediatric qualifiers						
No look back	54	51141	1.05	428598	1.26	
With look back	25	49452	0.51	412555	0.61	

^1^ Major Diagnostic Category.

^2^ Insufficient number of events to compare rates.

^3^ Length of stay. Categories are not exclusive.

*p < .001.

Spina bifida was identified as a risk factor when reviewing individual records of children who had a pressure ulcer. Therefore, the qualifying criterion in the paediatric algorithm that excluded children with paralysis in this study was revised to include ICD-10 codes for children with spina bifida (Table 1).

The 10 year look back was used to determine whether any of the children with pressure ulcer(s) recorded as a secondary diagnosis had a comorbid condition, such as a form of paralysis that should have excluded their pressure ulcer from being considered nursing sensitive. Of the 67 separations with pressure ulcer diagnoses recorded, seven (10.4%) had codes for a form of paralysis on their index record, and a further 29 (43%) had a form of paralysis recorded in previous separation records only (Table 4). Incorporating look back resulted in the detection of 0.51 nursing-sensitive pressure ulcers per 1,000 hospital separations; a rate of 0.61/10,000 patient days (Table 3).

**Table 4:** Paralysis identified in children with a pressure ulcer using a look back period

	**No of events**	**% of events**
Paralysis on same (index) separation	7	10.4
Paralysis on previous separations only	29	43.3
No paralysis recorded	31	46.3

### Pneumonia

Using Needleman et al.’s qualifying criteria [9] (Table 1), 413 records of nursing-sensitive pneumonia (3.86 per 1,000 hospital separations) were identified; a rate of 9.09/10,000 patient days (Table 5). Similar to Table 3, Table 5 shows the proportions and rates for various qualifying criteria. Numbers and rates of hospital separations with pneumonia are compared with and without cancer, immune deficiencies, community acquired pneumonia, respiratory system disorders and feeding difficulties. There were statistically significant differences between rates/10,000 patient days for qualifying criteria of those with and without community acquired pneumonia (95% CI 41.72, 96.06) and respiratory system disorders, coded MDC4 (95% CI 8.93, 15.48). There were <5 children with diagnoses of pneumonia and immune deficiency identified in the same separation (Table 5).

**Table 5:** Rates of pneumonia and analysis of qualifying criteria for pneumonia

	**No. of events**	**Total separations**	**No./1000 separations**	**Total patient days**	**Rate/10000 patient days**	**Diff between rates/10000 patient days**
						**95% CI**
As per Needleman	413	107026	3.86	454569	9.09	
**Qualifying criteria**						
Cancer						
With	11	1972	5.58	13789	7.98	(−8.12, 1.46)
Without	600	127747	4.70	530314	11.31	
Immune deficiencies						
With	<5	357	2.80	2087	4.79	−^1^
Without	>606	129362	4.72	542016	11.25	
Secondary diagnosis of community acquired pneumonia						
With	33	620	53.23	4146	79.59	(41.72, 96.06)*
Without	578	129099	4.48	539957	10.70	
MDC4^2^ (disorders of respiratory system)						
With	180	21341	8.43	83481	21.56	(8.93, 15.48)*
Without	431	108378	3.98	460622	9.36	
Feeding difficulties						
With	15	2374	6.32	13716	10.94	(−5.90, 5.31)
Without	596	127345	4.68	530387	11.24	
**Other pneumonia definitions**						
Ventilator associated	0	2708				
Postoperative	53	21592	2.45	129275	4.10	
Aspiration	94	129719	0.72	544103	1.73	
Epilepsy	26	2306	11.27	9896	26.27	(14.90, 35.10)*
No epilepsy	68	127413	0.53	534207	1.27	
Feeding difficulty	6	2374	2.53	13716	4.37	(−0.80, 6.23)
No feeding difficulty	88	127345	0.69	530387	1.66	
As per recommended paediatric qualifiers						
No look back	368	107141	3.43	455608	8.08	
With look back	337	102877	3.28	436271	7.72	

^1^ Insufficient number of events to compare rates.

^2^ Major Diagnostic Category.

*p < .001.

Specific causes of pneumonia were analysed separately. There were no records of ventilator associated pneumonia (VAP) coded in any diagnostic categories despite 381,316 hours of continuous ventilatory support being recorded in 2,708 hospital separations. Rates of postoperative pneumonia [24] and aspiration pneumonia are shown in Table 5.

Two groups of children at risk for aspiration pneumonia are those who have seizure activity or epilepsy and those with feeding difficulties [31]. There was a statistically significant difference in rates of aspiration pneumonia between those who had epilepsy (26.27/10,000 patient days) and those who did not (1.27/10,000 patient days) (difference 95% CI 14.90, 35.10). There were six children identified with aspiration pneumonia who had feeding difficulties, and the difference in rates was not significant (Table 5). Based on these results Needleman et al.’s algorithm was altered to include additional qualifying criteria, and the revised paediatric algorithm (Table 1) identified 3.43 records of pneumonia per 1,000 hospital separations; a rate of 8.08/10,000 patient days (Table 5).

The 10 year look back was used to determine whether any of the children with pneumonia recorded as a secondary diagnosis had a comorbid condition, such as the chronic respiratory conditions coded under MDC4, that should have excluded their pneumonia from being considered nursing sensitive. One patient had cystic fibrosis coded only on a previous separation and 13/611 had asthma only coded in previous separation records accounting for 2% of events. Exclusion of records with MDC4 on the index record accounted for most of the chronic respiratory conditions therefore using look back for further chronic respiratory conditions was not included in the revised algorithm (Table 6).

**Table 6:** Comorbid conditions identified in children with pneumonia using a look back period

	**No of events**	**% of events**
**Pneumonia**		
Asthma (not coded as MDC4^1^) on same separation	6	1.0
Asthma (not coded as MDC4) on previous separations only	13	2.1
No asthma or MDC4 recorded	592	96.9
Cystic fibrosis (not coded MDC4) on same separation	0	0.0
Cystic fibrosis (not coded MDC4) on previous separations only	1	0.2
No cystic fibrosis or MDC4 recorded	610	99.8
**Aspiration pneumonia**		
Epilepsy on same separation	26	27.7
Epilepsy on previous separations only	31	33.0
No epilepsy recorded	37	39.4

^1^ Major Diagnostic Category, disorders of respiratory system.

Data in the look back period were also reviewed for children with aspiration pneumonia recorded to ascertain whether previous diagnoses of epilepsy should have excluded their condition from being nursing sensitive. Thirty one hospital separations (33%) only recorded epilepsy prior to the index separation record of aspiration pneumonia (Table 6). Incorporating look back for epilepsy records resulted in the detection of 3.28 records of pneumonia per 1,000 hospital separations; a rate of 7.72/10,000 patient days (Table 5).

### Surgical wound infection

There were 122 surgical wound infections identified in secondary diagnoses from 21,592 surgical hospital separation records. The proportion of surgical wound infections as per Needleman et al.’s algorithm was 5.65/1,000 hospital separations. There were no exclusion criteria for this nursing-sensitive outcome in Needleman et al.’s algorithm. Assessment of whether having a compromised immune system or having cancer affected the number of events was undertaken, and indicated that no children who were immune compromised or had cancer had a surgical wound infection. This did not alter when data from the look back period was used (Table 7).

**Table 7:** Proportions of surgical wound infection and analysis of qualifying criteria for surgical wound infection

	**No. of events**	**Total separations**	**No./1000 separations**
As per Needleman	122	21592	5.65
**Qualifying criteria**			
Cancer^1^			
With	0	200	0.00
Without	122	21392	5.70
Immune deficiencies^1^			
With	0	31	0.00
Without	122	21561	5.66
Readmitted with surgical wound infection	103	21470	4.80
As per recommended paediatric qualifier	225	21592	10.42

^1^ Insufficient number of events to compare rates.

A look forward period of 30 days from date of surgical admission was used to ascertain whether any children classified as surgical were readmitted within WA with a surgical wound infection as a primary diagnosis. There were 103 children (4.79/1,000 hospital separations) who were readmitted to a WA hospital with a surgical wound infection without a diagnosis of wound infection in their previous surgical separation record. This gave 10.42 wound infections/1,000 surgical separations (Table 7).

## Discussion

In contrast to other nursing-sensitive outcome studies using administrative health data [10-12,24,32], this study used linked data, which enabled the researchers to identify all WA hospital separations during the previous 10 years for every child in the study cohort. The advantage of this data linkage is that comorbid conditions that are not recorded in the same hospital separation as the one that contained the outcome of interest can be identified. Using the linked data, 29 children with pressure ulcers (43% of all children with a pressure ulcer) were identified as also having a form of paralysis that was not identified in the same hospital separation as the pressure ulcer. Similarly, when using the look back period to identify children who had any forms of epilepsy which increased aspiration pneumonia risk, a further 31 (33%) children with aspiration pneumonia were excluded from the risk set. When records of children identified with these comorbid conditions are excluded from analysis the proportions and incidence rates of the nursing-sensitive outcomes are reduced, and more accurately quantify outcomes that reflect the quality of nursing care. However, the risk of excluding some nursing-sensitive outcomes remains.

The standard for coding additional diagnoses in hospital morbidity data is that the diagnosis must impact on patient care that requires ‘therapeutic treatment, diagnostic procedures or increased clinical care and/or monitoring’ [18] [p 13]. Older children hospitalised for treatment not related to a comorbid condition, such as spina bifida, cerebral palsy or epilepsy, are often self-caring in relation to their chronic condition; they don’t require an increase in resources, so these conditions are not coded in the index hospital separation abstract. The look back period in this study was 10 years preceding each child’s first separation from the tertiary hospital in the period 1999–2009, which for 77.3% of the children was from birth. Other researchers who have used linked data identified comorbid conditions either in the index separation record or they used a look back period, most frequently just 1 year [33]. For example, Preen et al. [17] used regression models to identify the impact of different comorbidity ascertainment look back periods and concluded that shorter periods of look back, approximately 1 year, were appropriate for post-hospitalisation mortality. However, it was suggested that longer look back periods were superior for other outcomes. The researchers reported that of the comorbid conditions recorded 5 years before the index hospitalisation, 46.8% were recorded at the index hospitalisation. This increased to 68.6%, 79.1% and 89.5% at 1, 2 and 3 years of look back respectively. The study was done with adult discharge abstracts and used 102 comorbid conditions identified in the Multipurpose Australian Comorbidity Scoring System (MACSS) [33]. Further work is required in this area, particularly in the paediatric population.

Using linked data was also beneficial for looking forward to ascertain children’s readmissions to hospitals in WA with a diagnosis of surgical wound infection. When the look forward period was included in the paediatric algorithm (Table 1), the proportion of surgical wound infections nearly doubled. The rationale suggested by Needleman et al. for including surgical wound infection as a nursing-sensitive outcome was related to nurses’ roles in preoperative preparation, which include skin cleaning and antibiotic administration [9]. However, the nurse’s role also includes postoperative assessment and monitoring, which should lead to early intervention to prevent wound infection. A principle of current paediatric care encourages home care as much as possible and results in shorter lengths of stay following surgery [34,35]. As surgical wound infections may not be noticed until a child has been discharged, it is reasonable to consider that children who present with a wound infection within a 30 day period of discharge from a surgical admission have an infection that is potentially nursing sensitive. Occurrence of wound infection could also reflect poor discharge planning with the child and their family regarding post-hospital wound care and medications. If parents are concerned about possible surgical wound infection in their child, children may return to the outpatient department, the surgeon’s private clinic, a general practitioner or emergency department and not require hospital admission with a surgical wound infection. These children are not accounted for in the current data linkage therefore rates are probably underestimating the true occurrence of infection.

Indicators of the quality of care may be used to compare rates of events across time or within and between units and hospitals. We were unable to ascertain whether the additional records with look back and look forward comorbid conditions were randomly distributed between hospitals or over time. If they were, calculations of rates would have consistent errors so including or excluding the extra cases would make little difference on relative performance when used for benchmarking.

As well as amending Needleman et al.’s algorithms to incorporate look back data for certain comorbid conditions, and look forward data for surgical wound infections, the algorithms were further tailored to suit paediatric populations by incorporating variations of qualifying criteria based on their impact in the study population. Differences between proportions or rates of events were analysed with and without each qualifying criterion applied for each nursing-sensitive outcome. For pressure ulcers statistically significant differences were found for each qualifier, except those with MDC9 (diseases of skin and subcutaneous tissues), where numbers were too low for meaningful analysis. Similarly, for pneumonia and surgical wound infection, numbers with immune deficiencies were too low; and for surgical wound infection, numbers with cancer were also too low for meaningful analysis. Although these qualifying criteria have not been included in our paediatric algorithms, regular reviews are recommended as the numbers of children with these diagnoses will alter with changes in treatments, diseases and coding.

Length of stay was analysed to ascertain the most appropriate duration qualifying criterion to apply when determining hospital-acquired pressure ulcers in children. There is a lack of consistency between researchers: some include adult hospital length of stay of longer than 3 days [9], others use longer than 4 days [36-39]. Curley et al. [40] found evidence of hospital acquired pressure ulcers in children as early as the second day of their hospital stay when assessing children in intensive care units during a point prevalence study. Number of pressure ulcers was low in our study when the length of stay criterion was less than 2 days, versus 2 or more days, so the statistical significance of the differences in rates using this criterion should be interpreted with caution. However, it is recommended that the paediatric algorithm for pressure ulcer include children who had a length of stay of longer than two days.

Patients with cancer are often immunocompromised and at increased risk of developing infections. Therefore, algorithms for nursing-sensitive outcomes that are the result of infections often include a qualifying criterion that removes this high risk group. The difference in rates of pneumonia in children with and without cancers was not statistically significant. As the rate was higher in children without cancer than those with cancer, this qualifying criterion was not included in the paediatric algorithm. Needleman et al. also removed it from their algorithm as it did not enhance the specificity of identifying outcomes in adult populations [9]. On the other hand, the criterion has been retained by others when calculating incidence of outcomes that are hospital acquired infections based on clinical reasoning [38,39]. The latest version of outcomes recommended by the Agency for Healthcare Research Quality (AHRQ) has not excluded children with cancers but stratified them into different risk groups [41]. In larger populations the number of children with cancer and pneumonia may be greater and stratification or exclusion of this group of children may be necessary.

As pneumonia is frequently used to indicate the quality of nursing care, [7,24,42] and the results of our earlier Delphi study [28] prioritised subgroups of pneumonia as nursing-sensitive outcomes, proportions and rates of three subgroups were calculated. The ICD codes for the individual pneumonia types were included in Needleman et al.’s algorithm. Ventilator associated pneumonia, which is particularly used to indicate the quality of care in intensive care settings, had no records coded despite children in the cohort requiring 381,316 hours of ventilation. Follow up with the coders confirmed that there were no records coded as they did not identify any diagnoses of ventilator associated pneumonia in the medical records (Logan, J. personal communication). Postoperative pneumonia, which is applicable to surgical patients only [24], appears to be a viable indicator of the quality of nursing care, but there were too few records of aspiration pneumonia in our cohort to evaluate it.

This cohort of hospitalised children is similar to cohorts of children in public hospitals across Australia. More boys than girls under 15 years of age are hospitalised [43] and the most common reasons for hospital admission are respiratory and gastrointestinal conditions in children aged less than 14 years, and rates of injury, poisoning and other external causes increase with age [44,45]. Other leading causes of hospitalisation are chronic diseases of tonsils and adenoids [45].

### Limitations

A limitation of using WA administrative health data for identifying nursing sensitive outcomes is the inability to determine whether secondary diagnoses are pre-existing comorbid conditions or complications that occurred during hospitalisation. The use of linked data can reduce this limitation by identifying children who have comorbid conditions recorded in previous separation records and can be excluded from being considered to have a nursing-sensitive outcome.

A further limitation is that the date of surgery was not included in HMD, so the 30 days post-surgery timeframe assumed that surgery occurred on day one of the hospitalisation. There is potential to have under estimated the rate of surgical wound infections as patients who had their surgery later than day one would not have had 30 days post–surgery included in the analysis. However, it may be more accurate than no outer time limit. [46,47] To increase the accuracy of this indicator, and others which measure quality postoperatively, it would be beneficial if the date of surgery became a routine variable in HMD.

There is a likelihood of under or over reporting of outcomes. Under reporting occurs particularly when the outcomes have no financial implication for the health service provider [9,48]. Not all nursing care is recorded in patient’s clinical records [25,27] and not all of the clinical records are coded into the separation records [18]. However, an audit of coding of ICD-10-AM showed a high level of reliability and adherence to coding standards [49]. Validation studies using review of hospital charts have found a reasonably high level of data accuracy and reported 87% accuracy for DRG coding within WA HMD [21]. The advantages of feasibility, cost saving and having complete longitudinal population data when using administrative health data offsets the limitations and can provide reliable population-based estimates of nursing-sensitive outcomes.

As numbers of nursing–sensitive outcomes are small in this WA paediatric population, comparisons of differences in rates may not be reliable, particularly when there are fewer than 5 records [50]. It is important to consider the number of records when interpreting results.

### Implications for research and policy

Using linked data is advantageous for identifying comorbidities that are not recorded on index separation records, but exclude children from being considered to have nursing-sensitive outcomes. When comorbidity records from previous separations are included, the specificity of the algorithm is increased, and a more accurate number of actual nursing-sensitive outcomes can be ascertained. Furthermore, linked data assists in identifying outcomes that become evident following discharge. When using administrative heath data, linked data should be used, particularly if all comorbid conditions are not routinely identified in the index separation record and to assist in the accurate identification of outcomes.

Numbers of pressure ulcers recorded in administrative health data in children are too few to be a useful measure of the quality of nursing care in this population. This finding is similar to results of a study undertaken in a large paediatric cohort in California [24]. However, the algorithms for pneumonia and surgical wound infections could potentially be considered for quality improvement initiatives within a hospital. As the health system and paediatric cohorts are similar across Australia [51], these algorithms are a starting point for national benchmarking to compare events and rates within tertiary paediatric hospitals. However, before being used for benchmarking between hospitals or areas within a hospital, additional risk factors must be identified to allow appropriate stratified analysis. Furthermore, paediatric algorithms can be used to determine whether there are associations between nursing-sensitive outcomes and levels of nurse staffing that would assist in ascertaining appropriate staffing levels in paediatric hospitals.

## Conclusion

Validation of algorithms prior to their use is an important step in the process of measuring nursing-sensitive outcomes in different patient populations. Changes need to be made to adult algorithms before applying them to paediatric cohorts. Using linked data is advantageous in enhancing the sensitivity of algorithms for nursing-sensitive outcomes.

## Competing interests

The authors declare that they have no competing interests.

## Authors’ contribution

SW conceived the study, secured funding, carried out the study design, data analyses, drafted the manuscript. AB assisted with data analyses, revised manuscript. YH revised manuscript. JF conceived the study, secured funding, assisted with data analyses, revised manuscript. All authors read and approved the final manuscript.

## Pre-publication history

The pre-publication history for this paper can be accessed here:

http://www.biomedcentral.com/1472-6963/12/209/prepub

## References

[B1] Nursing matters fact sheet: nursing sensitive outcome indicators [http://www.icn.ch/images/stories/documents/publications/fact_sheets/15c_FS-Nursing_Sensitive_Outcome_Indicators.pdf]

[B2] Yang K-PA Simms LM Yin J-CT Factors influencing nursing-sensitive outcomes in Taiwanese nursing homes Online J Issues Nurs 1999 4 2

[B3] Idvall E Rooke L Hamrin E Quality indicators in clinical nursing: a review of the literature J Adv Nurs 1997 25 6 17 10.1046/j.1365-2648.1997.1997025006.x 9004005

[B4] Aiken LH Clarke SP Cheung RB Sloane DM Silber JH Educational levels of hospital nurses and surgical patient mortality JAMA 2003 290 12 1617 1623 10.1001/jama.290.12.1617 14506121 PMC3077115

[B5] Aiken LH Clarke SP Sloane D Sochalski J Silber J Hospital nurse staffing and patient mortality, nurse burnout, and job dissatisfaction JAMA 2002 288 16 1987 1993 10.1001/jama.288.16.1987 12387650

[B6] Blegen M Goode C Reed L Nurse staffing and patient outcomes Nurs Res 1998 47 1 43 50 10.1097/00006199-199801000-00008 9478183

[B7] Needleman J Buerhaus P Mattke S Stewart M Zelevinsky K Nurse-staffing levels and the quality of care in hospitals N Engl J Med 2002 346 22 1715 1722 10.1056/NEJMsa012247 12037152

[B8] Tourangeau AE Doran DM Pringle D O’Brien-Pallas L McGillis Hall L Tu JV Verma A Nurse staffing and work environments: relationships with hospital level outcomes 2006 Toronto: University of Toronto 1 30

[B9] Needleman J Buerhaus PI Potter V Mattke S Stewart M Zelevinsky K Nurse staffing and patient outcomes in hospital 2001 Boston: Harvard School of Public Health i-487

[B10] McCloskey BA Diers DK Effects of New Zealand’s health reengineering on nursing and patient outcomes Med Care 2005 43 11 1140 1146 10.1097/01.mlr.0000182549.85761.cd 16224308

[B11] Duffield C Diers D O’Brien-Pallas L Aisbett C Roche M King M Aisbett K Nursing staffing, nursing workload, the work environment and patient outcomes Appl Nurs Res 2011 24 244 255 10.1016/j.apnr.2009.12.004 20974086

[B12] Twigg D Duffield C Bremner A Rapley P Finn J The impact of the nursing hours per patient day (NHPPD) staffing method on patient outcomes: a retrospective analysis of patient and staffing data Int J Nurs Stud 2011 48 5 540 548 10.1016/j.ijnurstu.2010.07.013 20696429

[B13] Bohensky M Jolley D Sundararajan V Evans S Pilcher D Scott I Brand C Data linkage: a powerful research tool with potential problems BMC Health Serv Res 2010 10 346 10.1186/1472-6963-10-346 21176171 PMC3271236

[B14] Bradley CJ Penberthy L Devers KJ Holden DJ Health services research and data linkages: issues, methods, and directions for the future Health Serv Res 2010 45 5p2 1468 1488 10.1111/j.1475-6773.2010.01142.x 21054367 PMC2965887

[B15] Jones P Elias P Administrative data as a research resource: a selected audit 2006 Warwick, England: Economic and Social Research Council and Warwick Institute for Employment Research 1 123

[B16] Jutte DP Roos LL Brownell MD Administrative record linkage as a tool for public health research Annu Rev Public Health 2011 32 91 108 10.1146/annurev-publhealth-031210-100700 21219160

[B17] Preen DB Holman CDAJ Spilsbury K Semmens JB Brameld KJ Length of comorbidity lookback period affected regression model performance of administrative health data J Clin Epidemiol 2006 59 9 940 946 10.1016/j.jclinepi.2005.12.013 16895817

[B18] National Centre for Classification in Health Australian coding standards 2008 6 Sydney: University of Sydney

[B19] Kelman CW Bass AJ Holman CDAJ Research use of linked health data: a best practice protocol Aust N Z J Public Health 2002 26 3 251 255 10.1111/j.1467-842X.2002.tb00682.x 12141621

[B20] Linkage process[http://www.datalinkage-wa.org/data-linkage/linkage-process]

[B21] Holman CDAJ Bass AJ Rouse IL Hobbs MST Population-based linkage of health records in Western Australia: development of a health services research linked database Aust N Z J Public Health 1999 23 5 453 459 10.1111/j.1467-842X.1999.tb01297.x 10575763

[B22] Shuldham C Parkin C Firouzi A Roughton M Lau-Walker M The relationship between nurse staffing and patient outcomes: a case study Int J Nurs Stud 2009 46 7 986 992 10.1016/j.ijnurstu.2008.06.004 18675419

[B23] Sochalski J Is more better? The relationship between nurse staffing and the quality of nursing care in hospitals Med Care 2004 42 Suppl 2 II-67 II-73 14734944 10.1097/01.mlr.0000109127.76128.aa

[B24] Mark BA Harless DW Berman WF Nurse staffing and adverse events in hospitalized children Policy Polit Nurs Pract 2007 8 2 83 92 10.1177/1527154407303499 17652626

[B25] Stratton KM Pediatric nurse staffing and quality of care in the hospital setting J Nurs Care Qual 2008 23 2 105 114 10.1097/01.NCQ.0000313758.33654.49 18344775

[B26] Beal AC Co JPT Dougherty D Jorsling T Kam J Perrin J Palmer RH Quality measures for children’s health care Pediatrics 2004 113 1 199 209 14702502

[B27] Lacey S Smith JB Cox KS Hughes RG From Chapter 15. Pediatric safety and quality Patient safety and quality: an evidence-based handbook for nurses 2008 Rockville, MD: AHRQ Publication No 1 30 08–0043

[B28] Wilson S Hauck Y Bremner A Finn J Quality nursing care in Australian paediatric hospitals: a Delphi approach to identifying indicators J Clin Nurs 2012 21 1594 1605 10.1111/j.1365-2702.2011.04004.x 22416966

[B29] Twigg D Duffield C Thompson PL Rapley P The impact of nurses on patient morbidity and mortality: the need for a policy change in response to the nursing shortage Aust Health Rev 2010 34 3 312 316 10.1071/AH08668 20797363

[B30] Horan TC Andrus M Dudeck MA CDC/NHSN surveillance definition of health care–associated infection and criteria for specific types of infections in the acute care setting Am J Infect Control 2008 36 5 309 332 10.1016/j.ajic.2008.03.002 18538699

[B31] Tai D Dick P To T Wright JG Development of pediatric comorbidity prediction model Arch Pediatr Adolesc Med 2006 160 3 293 299 10.1001/archpedi.160.3.293 16520449

[B32] Needleman J Buerhaus P Pankratz VS Leibson CL Stevens SR Harris M Nurse staffing and inpatient hospital mortality N Engl J Med 2011 364 11 1037 1045 10.1056/NEJMsa1001025 21410372

[B33] Holman CDAJ Preen DB Baynham NJ Finn JC Semmens JB A multipurpose comorbidity scoring system performed better than the Charlson index J Clin Epidemiol 2005 58 10 1006 1014 10.1016/j.jclinepi.2005.01.020 16168346

[B34] Australian Institute of Health and Welfare From Australian hospital statistics 2009–10 2011 Canberra: AIHW: Health services series no 40 edition

[B35] Care of children and adolescents in U.S. hospitals[http://www.ahrq.gov/data/hcup/factbk4/factbk4b.htm]

[B36] Pediatric quality indicators #2 technical specifications provider-level indicator pressure ulcer rate [http://www.qualityindicators.ahrq.gov/Downloads/Software/SAS/V43/TechnicalSpecifications/PDI%2002%20Pressure%20Ulcer%20Rate.pdf]

[B37] McDonald KM Davies SM Haberland CA Geppert JJ Ku A Romano PS Preliminary assessment of pediatric health care quality and patient safety in the United States using readily available administrative data Pediatrics 2008 122 2 E416 E425 10.1542/peds.2007-2477 18676529

[B38] Miller MR Zhan C Pediatric patient safety in hospitals: a national picture in 2000 Pediatrics 2004 113 6 1741 1746 10.1542/peds.113.6.1741 15173500

[B39] Sedman A Harris JM Schulz K Schwalenstocker E Remus D Scanlon M Bahl V Relevance of the Agency for Healthcare Research and Quality patient safety indicators for children’s hospitals Pediatrics 2005 115 1 135 145 15579669 10.1542/peds.2004-1083

[B40] Curley MAQ Quigley SM Lin M Pressure ulcers in pediatric intensive care: incidence and associated factors Pediatr Crit Care Med 2003 4 3 284 290 10.1097/01.PCC.0000075559.55920.36 12831408

[B41] Pediatric quality indicators #12 technical specifications provider-level indicator central venous catheter-related blood stream infection rate [http://www.qualityindicators.ahrq.gov/Downloads/Software/SAS/V43/TechnicalSpecifications/PDI%2012%20Central%20Venous%20Catheter-Related%20Blood%20Stream%20Infection%20Rate.pdf]

[B42] National Quality Forum (US) From Tracking NQF-endorsed consensus standards for nursing-sensitive care: A 15-month study 2007 Washington DC: National Quality Forum

[B43] Australian Institute of Health and Welfare Australian hospital statistics 2004–05 2006 Canberra: AIHW 370

[B44] Australian Institute of Health and Welfare Australia’s health 2010 2010 Canberra: AIHW

[B45] Health of children in Australia: A snapshot, 2004–05 http://abs.gov.au/ausstats/abs@.nsf/ProductDocumentCollection?OpenAgent&productno=4829.0.55.001&issue=2004-05

[B46] Agency for Healthcare Research and Quality (US) From Pediatric Quality Indicators #10 Technical Specifications Provider-Level Indicator Postoperative sepsis rate 2011 Rockville, MD: AHRQ

[B47] McDonald K Romano P Davies S Haberland C Geppert J Ku A Choudhry K From Measures of pediatric health care quality based on hospital administrative data 2006 AHRQ: the pediatric quality indicators

[B48] Lawthers AG McCarthy EP Davis RB Peterson LE Palmer RH Iezzoni LI Identification of in-hospital complications from claims data: is it valid? Med Care 2000 38 8 785 795 10.1097/00005650-200008000-00003 10929991

[B49] Henderson T Shepheard J Sundararajan V Quality of diagnosis and procedure coding in ICD-10 administrative data Med Care 2006 44 11 1011 1019 10.1097/01.mlr.0000228018.48783.34 17063133

[B50] Dawson B Trapp RG Basic and clinical biostatistics 2004 4 New York: Lange Medical Books/McGraw-Hill

[B51] Clark A Preen DB Ng JQ Semmens JB Holman CDAJ Is Western Australia representative of other Australian States and Territories in terms of key socio-demographic and health economic indicators? Aust Health Rev 2010 34 2 210 215 10.1071/AH09805 20497735

